# Effective coverage for hypertension treatment among middle-aged adults and the older population in China, 2011 to 2013: A nationwide longitudinal study

**DOI:** 10.7189/jogh.10.010805

**Published:** 2020-06

**Authors:** Yang Zhao, Ajay Singh Mahal, Shenglan Tang, Tilahun Nigatu Haregu, Brian Oldenburg

**Affiliations:** 1 The Nossal Institute for Global Health, The University of Melbourne, Melbourne, Australia; 2 WHO Collaborating Centre on Implementation Research for Prevention & Control of NCDs, Melbourne, Australia; 3 Duke Global Health Institute, Duke University, Durham, North Carolina, USA

## Abstract

**Methods:**

We used the baseline survey and first follow-up surveys of the China Health and Retirement Longitudinal Study of middle-aged and older populations conducted between 2011 and 2013. Correlates of effective coverage and treatment coverage for hypertension were analysed using multivariate logistic regression models, after controlling for demographic characteristics.

**Results:**

In 2011, 38.40% of 13 702 individuals surveyed were identified with hypertension. Overall, the effective treatment coverage among the middle-aged and older population in China from 2011 to 2013 was only 22.40% compared to the treatment coverage of 55.86%. Variations in effective coverage among patients enrolled in the three public health insurance schemes ranged from 22.60% to 29.31%.

**Conclusions:**

The level of effective coverage for hypertension treatment in China was still very low, and that health insurance schemes play a significant role in improving treatment coverage and effective coverage for hypertension treatment. In the implementation of China’s health system reform, health equity and health care equality should be emphasised and enhanced by offering more equitable benefits packages across social health insurance schemes.

In the context of sustainable development goals (SDG), advancing universal health coverage (UHC) represents the centrepiece of health policy in many countries [1]. A key goal is the reduction in premature mortality from non-communicable diseases (NCDs) by one-third by 2030, mainly through prevention and treatment [2]. Like most countries in the world, China has recently experienced a very rapid epidemiological transition from a preponderance of infectious disease to NCDs, including hypertension, diabetes, cardiovascular disease and cancers [3]. One illustration of the health challenges is the prevalence of hypertension having risen to around 44.7% among Chinese adults by 2017. In China, the number of people with hypertension has steadily increased over time [4]; however, only 35.7% of patients with hypertension were aware of their condition, and less than 18% of those people were effectively controlling their hypertension [5-7].

To cope with the challenges of NCDs in China, the National Public Health Initiative was instituted in 2009, promoting health care equity and strengthening access to primary public health services. It included improvements to disease screening and management of hypertensive individuals. The State Council of China also launched a comprehensive health system reform in 2009 to increase access to primary health care and to lower the financial burden of illness [8]. It resulted in a significant increase in health insurance coverage, with 95.7% of the Chinese population being covered by the three primary health insurance schemes in 2011 (that is, the Urban Employee Basic Medical Insurance (UEBMI), the Urban Resident Basic Medical Insurance scheme (URBMI), and the New Rural Cooperative Medical Scheme (NCMS)). Benefits packages and the degree of financial protection afforded to patients with NCDs varied across the three insurance schemes however [9].

Although previous studies have examined the prevalence, rates of awareness, treatment, and control of hypertension in China, most of them are based primarily on cross-sectional data sets [5,10-12]. Cross-sectional data used to estimate the effectiveness of treatment for hypertension has limitations as it does not provide information on changes in blood pressure over time for the same individual. Cross-sectional studies therefore cannot identify those hypertensive individuals with partially-controlled blood pressure. Longitudinal data on the other hand could be used to account for sub-optimally treated hypertensive patients with partial reductions in blood pressure over time, data which cannot be captured from cross-sectional analyses. In contrast to approaches that emphasise the sharp distinction between uncontrolled and controlled hypertensive cases, applying the metric of effective coverage has the advantage of estimating actual reductions in blood pressure due to intervention over time using longitudinal data sets, regardless of whether blood pressure reduction is below the well-controlled targets (for example, 90/140 mm Hg).

The differential impacts of reformed health insurance schemes on the management of hypertension since 2009 are not well understood, with previous work on this subject having limitations due to cross-sectional analysis. This study applies the metric of effective coverage to capture actual health changes in patients treated with anti-hypertensive medication over time. It considers patients with blood pressure above the standard thresholds of well-controlled hypertension. Furthermore, it investigates the relationship between indicators of hypertension treatment and demographic characteristics, health service use, and health system factors, particularly the three health insurance schemes.

## METHODS

### Sample and data

We used the national baseline and first follow-up surveys of the China Health and Retirement Longitudinal Study (CHARLS) conducted in 2011 and 2013. The CHARLS collected data from a nationally-representative sample of the Chinese population aged 45 and above for research on the elderly and on health trends. It was purposely designed to be consistent with and comparable to the Health and Retirement Study (HRS) in the United States. Funding support for the CHARLS mainly came from Peking University, the Behavioral and Social Research Division of the National Institute on Aging and from the World Bank. Written informed consent was obtained from all participants. CHARLS received ethics approval from the Peking University Biomedical Ethics Review Committee (Ref. No. IRB00001052-11015) in 2011 [13].

The CHARLS’ questionnaire covered the following key domains: demographics, health status and functioning, health care and insurance, work, retirement and pensions, income and consumption, household assets, and several biomarkers including height, weight, blood pressure, pulse rate, lung peak flow rate, and balance measures. Each respondent’s systolic blood pressure (SBP) and diastolic blood pressure (DBP) were recorded three times by a trained nurse using a HEM-7112 electronic monitor (OMRON, Tokyo, Japan). The average values for each participant were calculated but only given to the subjects once the interviews were completed. The interviewees were also asked if they had hypertension and whether they were receiving any form of antihypertensive treatment.

To ensure sample representativeness, the CHARLS baseline survey covered 150 counties/districts and 450 villages/urban communities across 28 provinces using multistage stratified probability-proportionate-to-size (PPS) sampling methods. In the first stage, 150 county-level units were randomly chosen with the PPS sampling method from a sampling frame containing all county-level units with the exception of those in Tibet and stratified by region and then by the district in urban areas or county in rural areas and by per capita gross domestic product. In the second stage, three primary sampling units (villages in rural areas or communities in urban areas) were randomly chosen within each county-level unit, using PPS sampling. Households living in the eligible dwellings were included, excluding empty or non-resident dwellings. Finally, the eligible member aged 45 and older and his or her spouse (if present) were interviewed. A total of 17 708 individuals in 10 257 households were successfully interviewed in the baseline CHARLS survey. The response rate was 80.5% in all age-eligible households in 2011, and 85.8% in 2013 among all baseline individuals. Ongoing follow-up surveys were conducted every two years. Further information on the methodology of the CHARLS is available from the online documentation [13].

After excluding cases with missing demographic information and blood pressure measurements, complete data were available for 13 702 individuals in 2011-12 and 12 897 individuals in 2013. A total of 5262 hypertensive individuals were identified in the baseline study. 1789 cases were excluded due to missing blood pressure values in the follow-up survey in 2013, and 126 cases excluded due to participant death. A total of 3347 participants were finally included and used to assess the extent of effective coverage for hypertension treatment and to perform a multivariate logistic regression analysis for identifying key correlates of effective coverage for hypertension treatment.

### Definition of hypertension and coverage indicators

In the current study, hypertension was defined as systolic blood pressure ≥140 mm Hg and/or diastolic blood pressure ≥90 mm Hg and/or being on medication for raised blood pressure [14]. Treatment coverage was defined as the percentage of hypertensive individuals who reported the use of antihypertensive medication.

Effective coverage is defined by the WHO as “the fraction of potential health gain that can be delivered to the population through a specific intervention or health system, given its capacity [15].” It can be measured for a single, or for multiple interventions, and/or for the whole health system [16]. In this study, we defined effective coverage of hypertension treatment (that is effective treatment coverage) as the fraction of actual blood pressure reduction achieved by taking antihypertensive medication from 2011 to 2013. The denominator was targeted reduction of blood pressure, namely the difference between participants’ blood pressure measured in 2011 and treatment targets (that is, 90/140 mm Hg).

The three critical components of effective coverage were operationalized as follows: 1) **Need,** refers to individuals with SBP≥140 mm Hg and/or DBP≥90 mm Hg measured in 2011; 2) **Use,** refers to the self-reported use of antihypertensive medication; and 3) **Quality**, refers to actual reduction in systolic blood pressure and/or diastolic blood pressure through taking antihypertensive medication from 2011 to 2013 as measured in the CHARLS surveys. For those cases where blood pressure increased over time, we calculated the quality of medication treatment to be zero, which means no reduction in blood pressure or no health gain for hypertensive patients; therefore, effective coverage for hypertension treatment was also calculated as zero, which means that the medication treatment for hypertension is ineffective, or the effect is zero. At the individual level, effective coverage for hypertension treatment estimates the fraction of blood pressure reduction that is delivered to the population who take the antihypertensive medication, as shown in the following equation:

*EC_i_* = (*Q_i_U_i_|N_i_* = 1)

Where *EC_i_* is the effective coverage of individual *i* with medication use; *Q_i_* is the expected quality of medication use as delivered to patient *i*; *U_i_* is the utilisation of antihypertensive medication among the population in need; and *N_i_* is an indicator of whether individual *i* needs the antihypertensive medication. At the population level, the overall effective coverage for a given intervention can also be aggregated, using each individual’s probability of effective coverage [15]. The conditional statement is important as it restricts *U_i_* to the condition of individual *i* who truly needs the intervention.

### Statistical analysis

Correlates of treatment coverage were analysed using multivariate logistic regression models, after controlling for demographic characteristics. An ordinal logistic regression model was applied to identify the correlates of effective coverage for hypertension. We divided the values of effective treatment coverage into three categories (1 = No change (effective coverage = 0); 2 = Some change (effective coverage was between 0 and 1); and 3 = effective change (effective coverage = 1)). The explanatory variables of interest were age, gender, household income per capita, level of education, marital status, region, location of residence, outpatient visits, hospitalisation, and type of health insurance scheme. The adjusted odds ratio (OR) and 95% confidence intervals (CI) were reported. We used STATA 14.0 (Stata Corp, College Station, TX, USA) for data analysis. *P* values less than 0.05 were considered statistically significant.

## RESULTS

### Sample characteristics

**Table 1** shows the descriptive statistics based on the final study population of 3347 respondents with complete data. The middle-aged and older population with hypertension contained a higher proportion of women (52%) than men (48%). In 2011, 96.81% of 3347 middle-aged and older Chinese adults with hypertension were covered by health insurance schemes, most of them enrolling in the New Cooperative Medical Scheme. Nearly 42% of those with hypertension were overweight or obese. **Table 2** shows that the mean SBP and DBP were 146.4mmHg and 84.4mmHg in 2011, and 143.6mmHg and 81.1mmHg in 2013, respectively. There were 39.78% of total hypertensive individuals with fully-controlled SBP, and 19.8% with partially-controlled SBP. Where reduction of DBP was required, 51.4% of hypertensive patients were fully-controlled, and 6.8% of patients were partially-controlled.

**Table 1:** Demographic characteristics of the study population

	Number	Unweighted %	Weighted %
Total:	3343	-	-
Age (years):			
45-54	853	25.52	26.35
55-64	1312	39.25	36.87
65-74	854	25.55	24.42
≥75	324	9.69	12.36
Gender:			
Male	1572	47.02	48.00
Female	1771	52.98	52.00
Marital status:			
Married	2844	85.07	83.26
Divorced/Widowed	473	14.15	15.77
Never married	26	0.78	0.97
Level of education:			
Illiterate	1021	30.54	28.34
Primary school	1385	41.43	40.11
Junior high school	638	19.08	19.98
Senior high school	260	7.78	9.63
College and over	39	1.17	1.94
Household income per capita (RMB):			
<500	555	16.6	14.68
500-1500	418	12.5	10.78
1500-4500	684	20.46	17.87
≥4500	1686	50.43	56.67
Health insurance:			
None	106	3.17	3.19
UEBMI	358	10.71	16.33
URBMI	173	5.17	6.82
NCMS	2633	78.76	71.13
Others	73	2.18	2.53
Residence location:			
Rural	2114	63.37	55.45
Urban	1222	36.63	44.55
Household registration:			
Agricultural	2682	80.37	72.83
Non-agricultural	655	19.63	27.17
Region:			
East	853	37.25	40.09
Central	906	39.56	37.53
West	531	23.19	22.39
BMI:			
<18.5	192	5.74	5.34
18.5-25	1851	55.37	52.87
25-30 (Overweight)	1041	31.14	33.69
≥30 (Obese)	259	7.75	8.11
Smoking:			
No	2339	70.28	71.59
Yes	989	29.72	28.41
Alcohol use:			
No	2301	68.83	68.16
Yes	1042	31.17	31.84

UEBMI – Urban Employee Basic Medical Insurance, URBMI – Urban Resident Basic Medical Insurance, NCMS – New Rural Cooperative Medical Scheme, Others – government health care, private medical insurance etc, BMI – body mass index

**Table 2:** Blood pressure and changes among hypertensive individuals in China, 2011-2013

Blood pressure	Unweighted		Weighted	
**Mean**	**SE**	**Mean**	**SE**
Systolic blood pressure:				
SBP in the year 2011	146.50	21.48	146.40	0.48
SBP in the year 2013	142.71	21.97	143.55	0.91
Reduction of SBP 2011-13	-3.79	25.05	-3.23	0.60
% of fully controlled SBP	39.75	-	39.46	-
% of partially controlled SBP	19.26	-	19.77	-
Diastolic blood pressure:				
DBP in the year 2011	84.62	14.56	84.42	0.33
DBP in the year 2013	80.79	12.31	81.13	0.49
Reduction of DBP 2011-13	-3.83	15.80	-3.57	0.35
% of fully controlled DBP (%)	52.44	-	51.42	-
% of partially controlled DBP (%)	5.98	-	6.75	-

SE – standard error, SBP – systolic blood pressure, DBP – diastolic blood pressure

### Health services for people with hypertension

In terms of utilisation of health service, **Table 3** reveals that both outpatient and inpatient care among patients with hypertension increased between 2011 and 2013 in China. During the period of 2011-13, the proportion of outpatient visit (and hospital admission) raised from 19.02% (10.08%) to 22.61% (14.68%). Similarly, the times of outpatient visit in last month and days of hospital stay in last year increased from 0.43 and 0.13, to 0.56 and 0.23, respectively. The proportion of physical examinations performed in the last year was 34.02% in 2011 and up to 50.18% in 2013. Moreover, a larger proportion of hypertensive persons was given health education or advice by health care providers in 2013 than in 2011. Health education for hypertensive patients included weight control, exercise, healthy diet, and smoking control advice.

**Table 3:** Health services for patients with hypertension in China between 2011 and 2013

Health services	Year 2011		Year 2013	
**Unweighted**	**Weighted**	**Unweighted**	**Weighted**
Proportion of outpatient visit	19.55%	19.02%	23.57%	22.61%
Number of outpatient visit (Mean/SE)	0.43 (0.025)	0.43 (0.035)	0.57 (0.027)	0.56 (0.046)
Admission rate	10.44%	10.08%	16.11%	14.68%
Days of hospital stay (Mean/SE)	0.14 (0.008)	0.13 (0.013)	0.26 (0.014)	0.23 (0.019)
Physical examination last year	25.10%	34.02%	35.19%	50.18%
Health education provided*	29.70%	41.79%	28.51%	45.99%

SE – standard error

*Health education includes weight control, exercise, healthy diet, and aspect of smoking control.

### Coverage of hypertension treatment

**Table 4** shows that overall effective coverage for hypertension treatment among the middle-aged and older population in China between 2011 and 2013 was 22.40%. It was much less than the overall treatment coverage of hypertension (55.86%). Hypertensive individuals who reported hypertension treatment in two survey rounds had higher effective coverage than those who only reported treatment in one survey round. Compared with patients younger than 65 years old, senior elders had a lower level of effective coverage. Female individuals with hypertension also had higher treatment coverage and effective coverage (62.42%, 24.16%) than male patients (48.68%, 20.48%).

**Table 4:** Effective coverage and treatment coverage for hypertension between Chinese population groups, 2011- 2013

	Effective coverage		Treatment coverage	
**Unweighted**	**Weighted**	**Unweighted**	**Weighted**
Overall	24.18%	22.40%	56.39%	55.86%
Age (years):				
45-54	26.58%	23.12%	52.64%	54.16%
55-64	27.94%	27.91%	61.13%	61.27%
65-74	18.97%	16.56%	54.45%	50.71%
≥75	16.34%	16.70%	52.16%	54.78%
Gender:				
Male	22.48%	20.48%	51.15%	48.68%
Female	25.69%	24.16%	61.04%	62.42%
Household registration:				
Agricultural	23.74%	23.23%	55.15%	53.71%
Non-agricultural	25.90%	20.21%	61.22%	61.28%
Level of education:				
Illiterate	23.11%	22.25%	55.83%	55.12%
Primary school	23.41%	20.78%	57.24%	54.33%
Junior high school	26.26%	27.92%	56.51%	57.73%
Senior high school	26.74%	22.65%	53.67%	50.99%
College and over	26.36%	10.47%	51.28%	79.48%
Household income per capita (RMB):				
<500	22.74%	21.09%	53.15%	49.47%
500-1500	21.75%	22.57%	53.35%	54.62%
1500-4500	25.12%	24.50%	60.23%	58.79%
≥4500	24.87%	22.04%	56.64%	56.81%
Health insurance:				
None	26.50%	18.41%	59.22%	56.99%
UEBMI	28.96%	29.31%	65.32%	68.40%
URBMI	23.48%	22.60%	55.53%	54.21%
NCMS	23.54%	24.04%	57.53%	63.77%
Others	26.36%	26.32%	52.83%	57.37%
Region:				
East	25.17%	23.61%	70.81%	74.65%
Central	28.09%	27.07%	73.40%	71.35%
West	25.07%	23.94%	66.10%	63.94%
BMI:				
<18.5	13.22%	10.50%	29.69%	33.39%
18.5-25	21.57%	20.80%	50.24%	48.52%
25-30 (Overweight)	29.24%	25.05%	67.92%	66.95%
≥30 (Obese)	30.59%	30.63%	73.75%	72.96%
Smoking				
No	25.83%	23.94%	60.15%	59.19%
Yes	20.18%	18.59%	47.52%	47.66%
Alcohol use:				
No	25.60%	23.41%	59.71%	60.29%
Yes	21.03%	20.31%	49.04%	46.64%

UEBMI – Urban Employee Basic Medical Insurance, URBMI – Urban Resident Basic Medical Insurance, NCMS – New Rural Cooperative Medical Scheme, Others – government health care, private medical insurance etc, BMI – body mass index

Considerable variations in effective coverage for hypertension treatment were observed between patients without health insurance and those with insurance, ranging from 18.41% to 29.31%. Meanwhile, there were differences in treatment coverage across insurance schemes, ranging from 54.21% to 68.40%. Furthermore, patients with current smoking, alcohol use, and low body mass index (BMI) had relatively low effective coverage for hypertension treatment (**Table 4**).

### Multivariate regression analysis

**Table 5** reports the results of multivariate regression analysis, showing that both treatment coverage and effective coverage for hypertension were directly correlated with health insurance, outpatient visit, hospitalisation, gender and residence region. After adjustment for key covariables, effective treatment coverage was significantly higher among patients who were in the UEBMI scheme (odds ratio (OR) = 1.903; 95% confidence interval (CI) = 1.259-2.876), who visited the doctor or other health provider during the previous month (OR = 1.838; 95% CI = 1.503-2.249), who were hospitalised in the past year (OR = 1.572; 95% CI = 1.211-2.042), and who reported living in the eastern region (OR = 1.416; 95% CI = 1.159-1.729) or central region in China (OR = 1.551; 95% CI = 1.274-1.888).

**Table 5:** Multivariate logistic regression analysis, impact of health insurance schemes on effective coverage and treatment coverage for hypertension

	Effective coverage*				Treatment coverage			
**OR**		**95% CI**		**OR**	**95% CI**		
Health insurance (None)	1				1			
UEBMI	1.903†	1.259		2.876	2.301‡		1.543	3.432
URBMI	1.332	0.845		2.099	1.658†		1.084	2.536
NCMS	1.433§	1.023		2.006	1.405§		1.032	1.912
Others	1.943†	1.113		3.391	1.944†		1.129	3.347
Outpatient visit last month (No)	1				1			
Yes	1.838‡	1.503		2.249	2.390‡		1.919	2.976
Hospitalization last year (No)	1				1			
Yes	1.572†	1.211		2.042	2.459‡		1.830	3.305
Gender (Male)	1				1			
Female	1.189§	1.010		1.400	1.439‡		1.229	1.686
Age (years, 45-54)	1				1			
55-64	1.221	0.997		1.495	1.423‡		1.170	1.729
65-74	1.103	0.877		1.386	1.202		0.965	1.498
≥75	0.894	0.652		1.226	1.145		0.848	1.545
Household income per capita (<500):	1				1			
500-1500	0.884	0.729		1.071	0.957		0.797	1.149
1500-4500	1.143	0.928		1.408	1.192		0.970	1.464
≥4500	1.055	0.841		1.323	1.169		0.938	1.458
Education (Illiterate):	1				1			
Primary school	1.210§	1.000		1.465	1.203§		1.002	1.443
Junior high school	1.444†	1.128		1.849	1.326†		1.041	1.689
Senior high school	1.063	0.753		1.502	1.190		0.855	1.656
College and over	1.225	0.646		2.323	1.124		0.594	2.128
Marital status (Never married):	1				1			
Married	0.842	0.346		2.046	1.497		0.605	3.705
Divorced/Widowed	0.847	0.340		2.108	1.371		0.542	3.469
Region (West):	1				1			
East	1.416†	1.159		1.729	1.520‡		1.259	1.837
Central	1.551‡	1.274		1.888	1.623‡		1.346	1.958
Residence location (Rural):	1				1			
Urban	1.036	0.872		1.231	1.023		0.866	1.209

CI – confidence interval, OR – odds ratio, UEBMI - Urban Employee Basic Medical Insurance, URBMI – Urban Resident Basic Medical Insurance, NCMS – New Rural Cooperative Medical Scheme, Others – government health care, private medical insurance, etc.

*Results of the ordinal logistic regression model.

†*P* < 0.05.

‡*P* < 0.01.

§*P* < 0.1.

## DISCUSSION

This paper applied the WHO concept of effective coverage to estimate the effectiveness of hypertension treatment in China, using a nationally representative sample of Chinese adults. We also identified the variations in treatment coverage (defined as the percentage of individuals using antihypertensive medication) and effective treatment coverage (defined as the fraction of actual blood pressure reduction through taking antihypertensive medication) across different Chinese population groups. Our findings revealed a large gap between treatment coverage and effective treatment coverage among middle-aged and older people with hypertension in China. This study also identified significant differences in effective coverage for hypertension treatment across age groups, health insurance schemes, and patients’ BMI levels.

### Treatment coverage and effective coverage for hypertension

As China seeks to further improve the health system’s effectiveness, the measurement of effective coverage of interventions/health care can serve as an essential performance indicator, a more useful indicator than the more usual estimation of treatment coverage (rate of medication treatment). This study revealed that there was relatively high treatment coverage in relation to the use of antihypertensive medications in China. The utilisation of outpatient services and inpatient care among hypertensive individuals also increased from 2011 to 2013. The new round of health system reform launched in 2009 played a significant role in promoting this trend via policies aimed at strengthening primary health care, improving access to essential medicines, and ensuring equitable utilisation of public health services; however, our findings also suggested that effective coverage for hypertension treatment was still quite low in China [4,5,10-12]. This finding was similar to the estimate of effective treatment coverage of hypertension in Mexico between 2005 and 2006 [17].

### Variations in treatment coverage and effective coverage

Large variations in both effective coverage and treatment coverage for hypertension exist across gender, age groups, health insurance schemes, and BMI levels of Chinese adults. Females with hypertension had a higher treatment coverage for antihypertensive care than males. Among the elderly, compared with the early older ages (45-64 years), senior elderly people (above 65 years) with hypertension were less likely to experience effective treatment coverage and controlled hypertension, despite similar treatment coverage. There might be physiological or age-related factors influencing the control of hypertension, such as arteriosclerosis, which is common among the elderly population [18].

Considerable variations in both treatment coverage and effective coverage for hypertension treatment also exist across the three public health insurance schemes, that is, the Urban Employee Basic Medical Insurance, the Urban Resident Basic Medical Insurance, and the New Rural Cooperative Medical Scheme. The differences in benefits packages and degree of financial protection across insurance schemes could lead to unequal utilisation of health care, and even to different health outcomes. Despite the achievement of having 95.7% of the Chinese population covered by insurance schemes in 2011 [9], the per capita annual fund contribution for UEBMI is approximately six times higher than that for URBMI, and seven times for NCMS [19]. The reimbursement rate is 10% lower, and health care coverage is also smaller for NCMS than for the other two insurance schemes [20]. This finding is consistent with several studies that suggested gradients in benefits packages associated with insurance schemes would lead to inequity in health services’ provision and risk protection [21,22], especially for people with NCDs [9].

### Key correlates of treatment coverage and effective coverage

In terms of the potential correlates, there are several similarities and differences between the results of this study and the findings of previous studies. Our findings showed a positive association between health insurance scheme and both treatment coverage and effective coverage of hypertension care. Some longitudinal studies in both developing and developed countries have indicated positive effects of health insurance on awareness and control of hypertension [23,24]. Consistent with large numbers of studies, there was a positive effect of health insurance on treatment coverage of hypertension [23,25-29]; however, Liao and colleagues suggested that health insurance had little effect on control of hypertension, based on findings from the China Health and Nutrition Survey (CHNS), a repeated cross-sectional design study from 1991 to 2009 [29]. One of the limitations of CHNS is its high proportion of missing data related to health insurance. Another limitation is the lack of information on measurement of participants' blood pressure, which might vary by survey waves and provinces. The conflicting results of CHARLS and the CHNS could also be due to the different metrics used. We applied the indicator of effective coverage for hypertension rather than the rate of well-controlled hypertension, which may ignore patients with only partial reduction in blood pressure.

There is no doubt that the current benefits packages are different across the three public health insurance schemes in China [14]. These differences could have an impact on health care utilisation, including the use of antihypertensive medication, blood pressure examination, and public health services provided by health professionals, such as health education in relation to physical exercise, healthy diet and smoking control. As shown by Xinglin Feng et al. [30], compared with other hypertensive individuals, people whose insurance schemes covered the costs of outpatient service are more likely to receive care and to be well-controlled. Generally, most NCDs, including hypertension, require relatively long periods of outpatient care [29]. Previous studies indicate however that current benefits packages are limited, mainly focusing on the costs of inpatient care [31]. Outpatient care for patients with NCDs has low reimbursement levels, so the financial support received may be inadequate [32].

This study showed that outpatient and inpatient service use had a positive correlation with antihypertensive medication treatment and effective coverage of hypertension care. The utilisation of both outpatient and inpatient services increased for Chinese people with hypertension over time. This pattern of health care utilisation in China is consistent with the expectations of policies implemented and reflects the intended outcomes of health system reform. Experiences from several developing countries have also demonstrated the relationship between effective treatment coverage and the availability, accessibility, acceptability, and quality of human resources for health [33]. Expansion of health service coverage results in increased use of antihypertensive medication and better control of blood pressure, particularly in places with a better supply of health professionals. Like China, access to hypertension care also varies by insurance status in Mexico. Hypertensive patients insured through Mexico's Seguro Popular have a high probability of receiving antihypertensive treatment and better blood pressure control [17].

Our results have important policy and research implications for China and other developing countries. We would expect that both treatment coverage and effective coverage of NCD care could be significantly improved through comprehensive health system reform, yet effective treatment coverage of hypertension has not improved as expected in China. Significant health disparities and unacceptably low effective coverage of hypertension care should be given more attention by China’s central and local governments [34]. Reducing disparities in health service utilisation and health gain could be used as key indicators for assessing the performance of China's health insurance reform and the National Public Health Initiative. Alternatively, effective measures could be considered to promote universal health coverage via offering equitable benefits package and fair financial risk protection, including an equal level of fund contributions, the same reimbursement rates, and more services covered, in the integration process of the three public health insurance schemes in China. For developing countries with economic transition, a crucial step towards achieving the goal of UHC will be strengthening the stewardship role of the health sector through better information and comprehensive health care reform to achieve effective regulation and introduce an ongoing evaluation process of the health system’s performance, with a focus on health care quality. Analyses of effective coverage would provide a more useful and appropriate indicator in monitoring the performance of the health care system, encouraging decision-makers to focus on the quality of service provision rather than only on accessibility or availability of health services. With the imminent need to monitor UHC in the SDG era, further analysis of effective coverage of treatment for severe chronic diseases and NCD management should be a very critical area of future research in China and other countries.

In terms of this study’s limitations, first, data on hypertension treatment and health service use was self-reported. This may be subject to recall bias. Second, due to the CHARLS data being based on a middle-aged and older population, effective coverage for hypertension treatment among younger Chinese adults (<45 years older) was not analysed. Third, around 20% of respondents had missing blood pressure values. We conducted adjusted analysis by using the created weights provided by the CHARLS team to address the problem of non-response bias. Finally, the relatively low proportion of hypertensive individuals without any health insurance able to serve as a comparison group may have affected the reliability of t treatment effectiveness estimates for hypertension.

## CONCLUSIONS

Given a steady increase in the number of individuals with hypertension in China, the level of effective coverage for hypertension treatment was very low at the time of data collection. As health insurance schemes play a significant role in improving effective treatment coverage of hypertension, China’s insurance reforms need to give further attention to this public health challenge. Equity in service utilisation and health care outcomes should be emphasised and enhanced by offering more equitable benefits packages across the three health insurance schemes in China. Moreover, to encourage decision-makers to focus on the quality of service provision rather than only service accessibility, further studies about effective coverage of treatment for other chronic diseases are needed.
